# Intrathoracic Schwannoma With Horner Syndrome: A Rare Association

**DOI:** 10.7759/cureus.77453

**Published:** 2025-01-14

**Authors:** John Khor, Diong Nguk Chai

**Affiliations:** 1 Internal Medicine, Sultanah Nora Ismail Hospital, Batu Pahat, MYS; 2 Thoracic Surgery, Hospital Sultan Ismail, Johor Bahru, MYS

**Keywords:** horner syndrome, intrathoracic, schwannoma, thoracic radiology, vats

## Abstract

An incidental large mediastinal mass on the left hemithorax was noted in the chest radiograph of a 21-year-old male with a history of active smoking (five pack years) after a manual reduction for left shoulder dislocation. Horner syndrome (HS) was elicited from history and physical examination. A contrast computed tomography (CT) of the thorax showed a large (8.1 x 7.3 x 7.3 cm), well-defined, heterogeneous enhancing mass over the left apical-posterior mediastinum. The CT-guided biopsy showed spindle cells, suggestive of lung neoplasm. Initial concerns were thus present for lung malignancy and he was referred to thoracic surgery for further evaluation.

The thoracic team decided on a left video-assisted thoracoscopic surgery (VATS) and the tumor was resected uneventfully. Unfortunately, the Horner syndrome persisted. The final histopathology confirmed schwannoma. He was briefed regarding the benign prognosis and was eventually discharged. This study serves to illustrate the incidental finding of schwannoma, a rare diagnosis, and its association with Horner syndrome.

## Introduction

A diameter of 3 cm is a cut-off between lung nodule and lung mass when seen in a chest radiograph (CXR). In most cases, a lung mass warrants further investigations as most are cancerous [1]. Horner syndrome (HS) further lends weight to this suspicion, especially when constitutional symptoms are reported. This eponymous condition is said to occur when there is damage or disruption in the sympathetic nerve supply along the first-order neuron (intracranial conditions), second-order neuron (intrathoracic condition), and third-order neuron (carotid artery) [2].

Schwannomas originate from Schwann cells and are the most common neurogenic tumors found in adults. They comprise 20% of all mediastinal neoplasms, typically affecting adults in their 30s-40s; although a wide range of ages (from six to 78 years) have been reported [2]. While the auditory cranial nerve VIII is the most common site affected, schwannoma can potentially affect any peripheral or cranial nerve cells in the human body. Fortunately, it is normally benign and slow-growing, exerting its effects only when adjacent organs are compressed. Afflicted patients are generally asymptomatic, and the tumor is often detected incidentally when thoracic imaging is done for other purposes. It is typically a well-circumscribed mass found in the mediastinum on chest radiograph (CXR) or a paraspinal encapsulated homogeneous or heterogeneous mass displacing adjacent structures without direct infiltration on the thorax's computed tomography (CT). Magnetic resonance imaging (MRI) of the thorax is also valuable in evaluating the potential invasiveness of the tumor. It may assist in identifying mediastinal or vascular abnormalities such as aortic aneurysms [3].

The relationship between HS and intrathoracic schwannoma is rarely reported, with only a handful of cases reported in the literature [4-7]. Differentiating between schwannoma and lung malignancy is important as prognosis and treatment direction differ remarkably. An aggressive course of therapy is indicated for the latter, while watchful waiting with surveillance or resection suffices for the former. A proper diagnosis allows for appropriate reassurances to alleviate any potential anxiety, particularly in smokers.

## Case presentation

**Figure 1:**
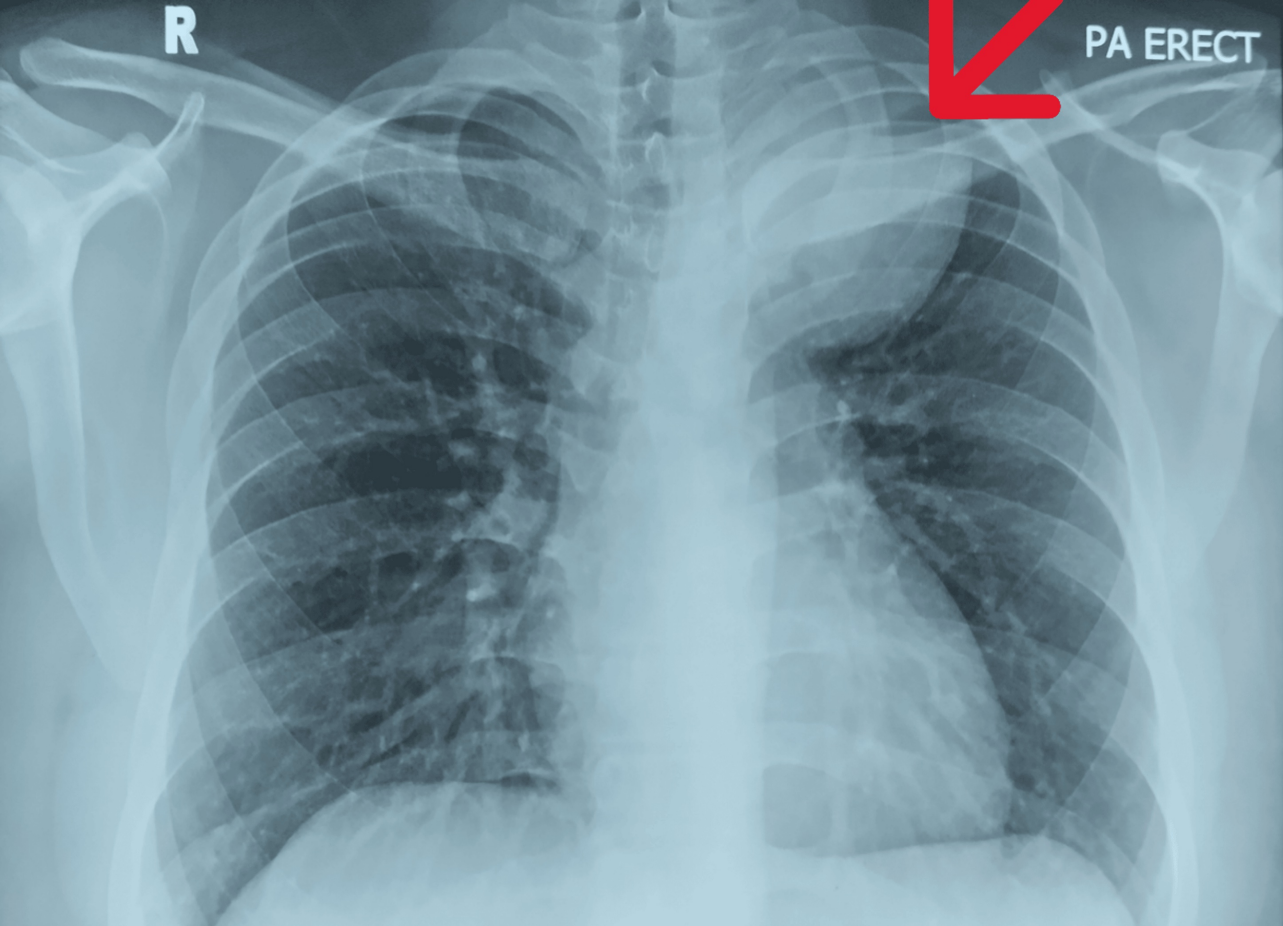
CXR post shoulder reduction. The arrow shows the rounded well-defined mass at the apical left hemithorax. CXR: chest radiograph

**Figure 2:**
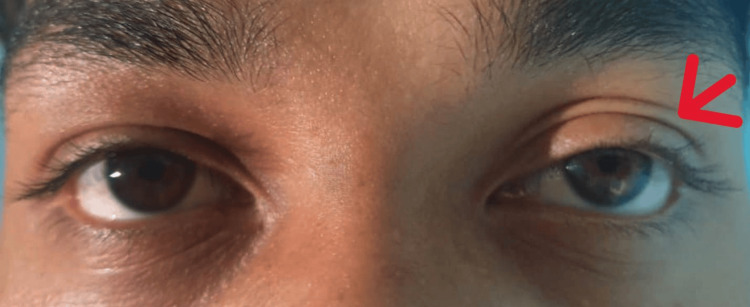
Horner syndrome (ptosis and miosis) seen in left eye (arrow).

A 21-year-old man, an active smoker of 20 sticks/day (five pack years), presented to our emergency department after a road traffic accident. He dislocated his right shoulder, necessitating manual reduction. An incidental finding of a left upper zone mass was noted on the post-reduction CXR (Figure 1). Further history and physical examination revealed Horner syndrome (HS), characterized by ipsilateral anhidrosis of the face, axilla, and palm; miosis; and partial ptosis, which had been present for more than four years (Figure 2). He denies any respiratory or constitutional symptoms and was generally well.

**Figure 3:**
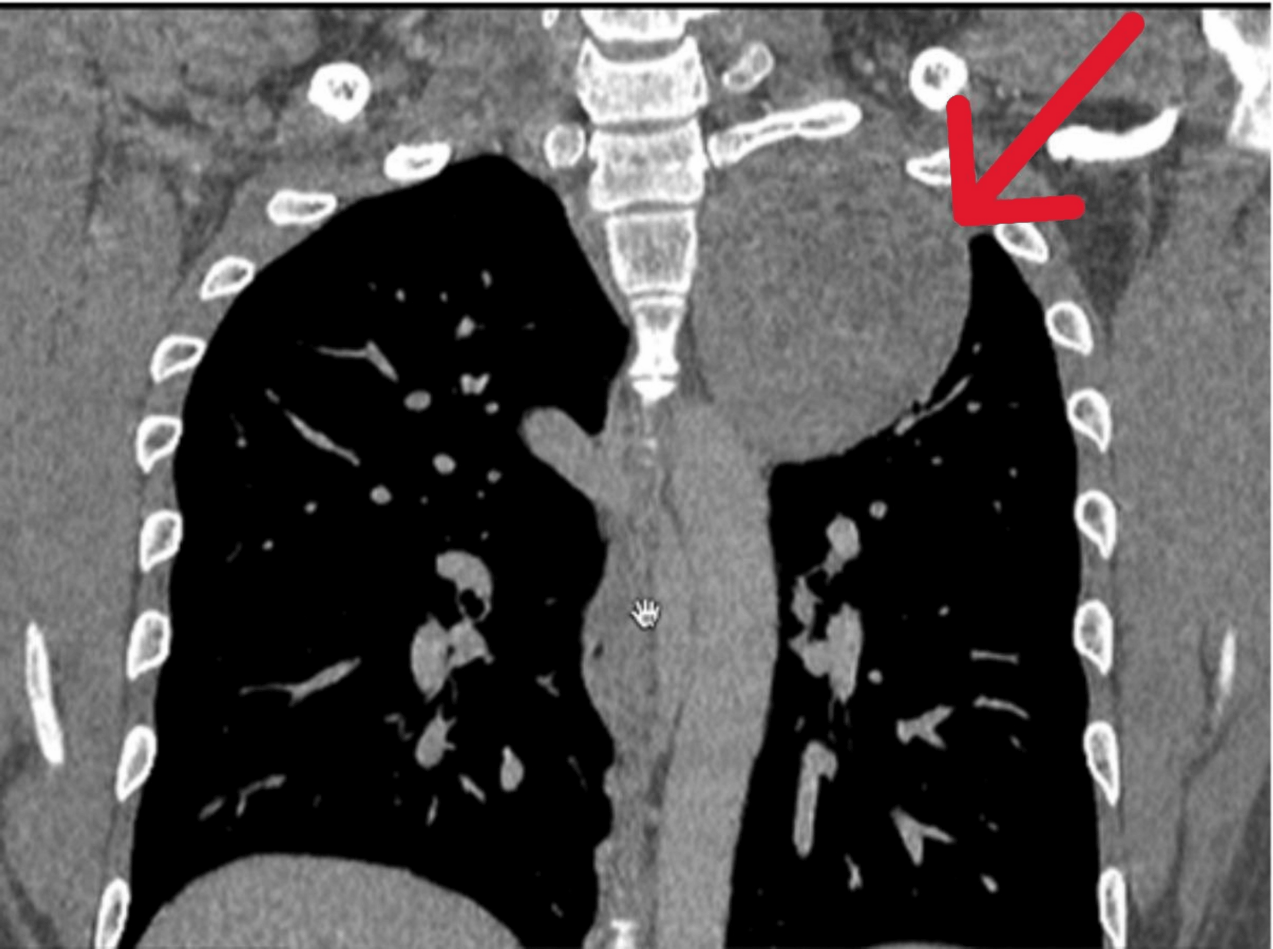
Coronal view of contrast-enhanced computed tomography (CECT) of thorax. The arrow shows the thoracic mass over the left upper region (coronal view).

Computed tomography (CT) of the thorax revealed an 8.1 x 7.3 x 7.3 cm heterogeneous well-circumscribed round paraspinal mass occupying the apico-posterior region of the left hemithorax (Figure 3).

**Figure 4:**
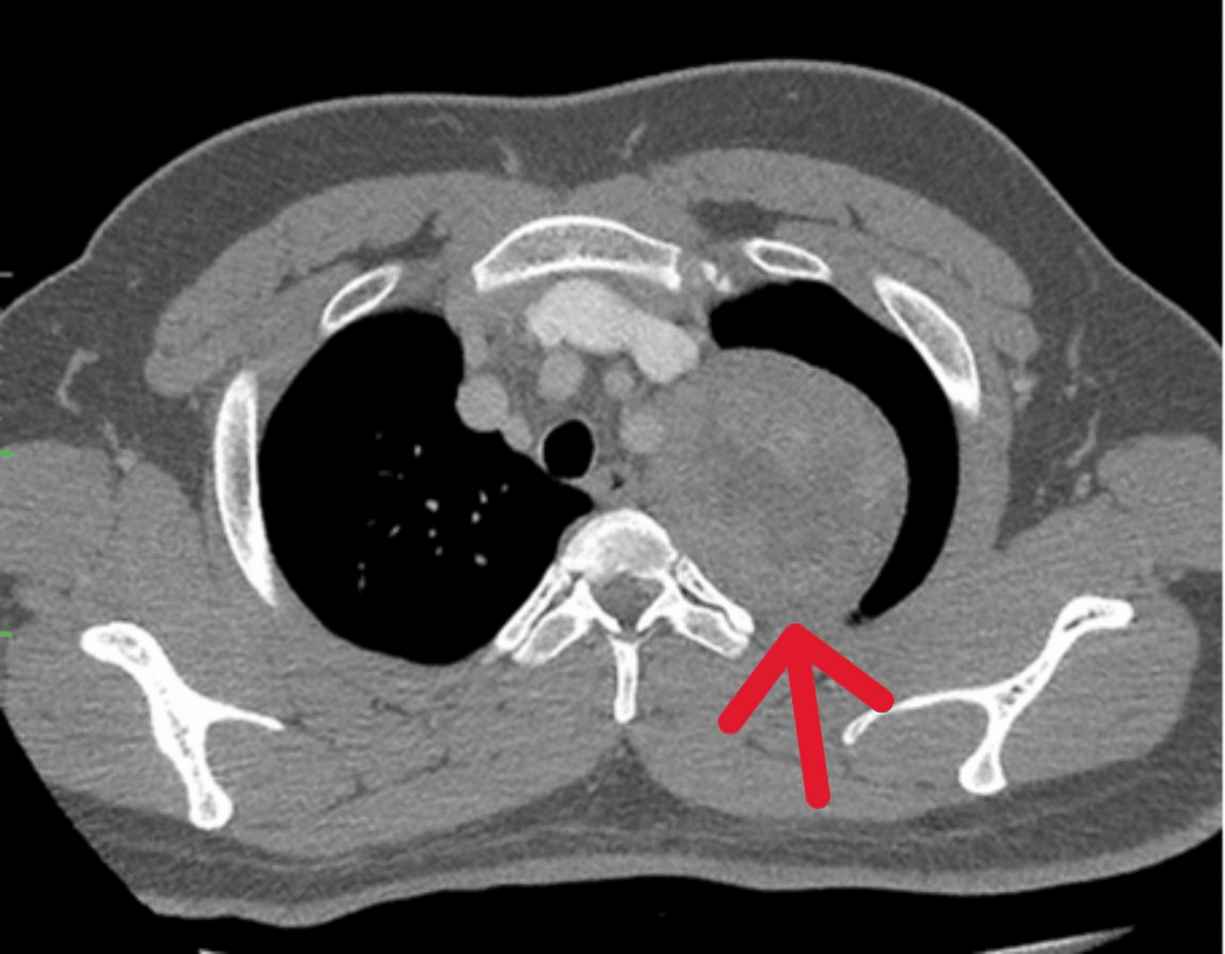
Axial view of contrast enhanced computer tomography (CECT) of thorax. The arrow shows the thoracic mass over the left upper region (cross-sectional view), the likely direction of the needle biopsy.

**Figure 5:**
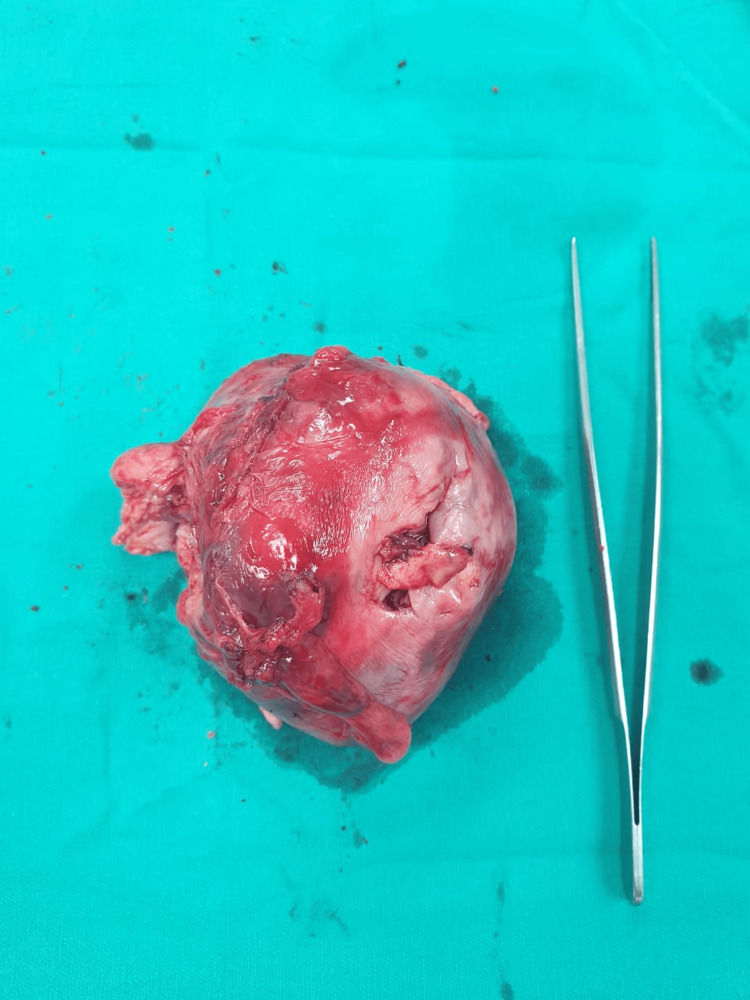
Gross inspection of the resected mass.

The tumor was adherent to the posterior first and second ribs and left subclavian artery (Figure 4). A CT-guided biopsy revealed spindle cell neoplasm with minimal atypia. He underwent left uniportal video-assisted thoracoscopic surgery (VATS) and resection of the tumor. A well-encapsulated left paraspinal tumor measuring 7.0 x 6.5 x 5.0 cm was excised completely (Figure 5). His post-operative recovery was uneventful, but the Horner syndrome persisted for up to two months.

**Figure 6:**
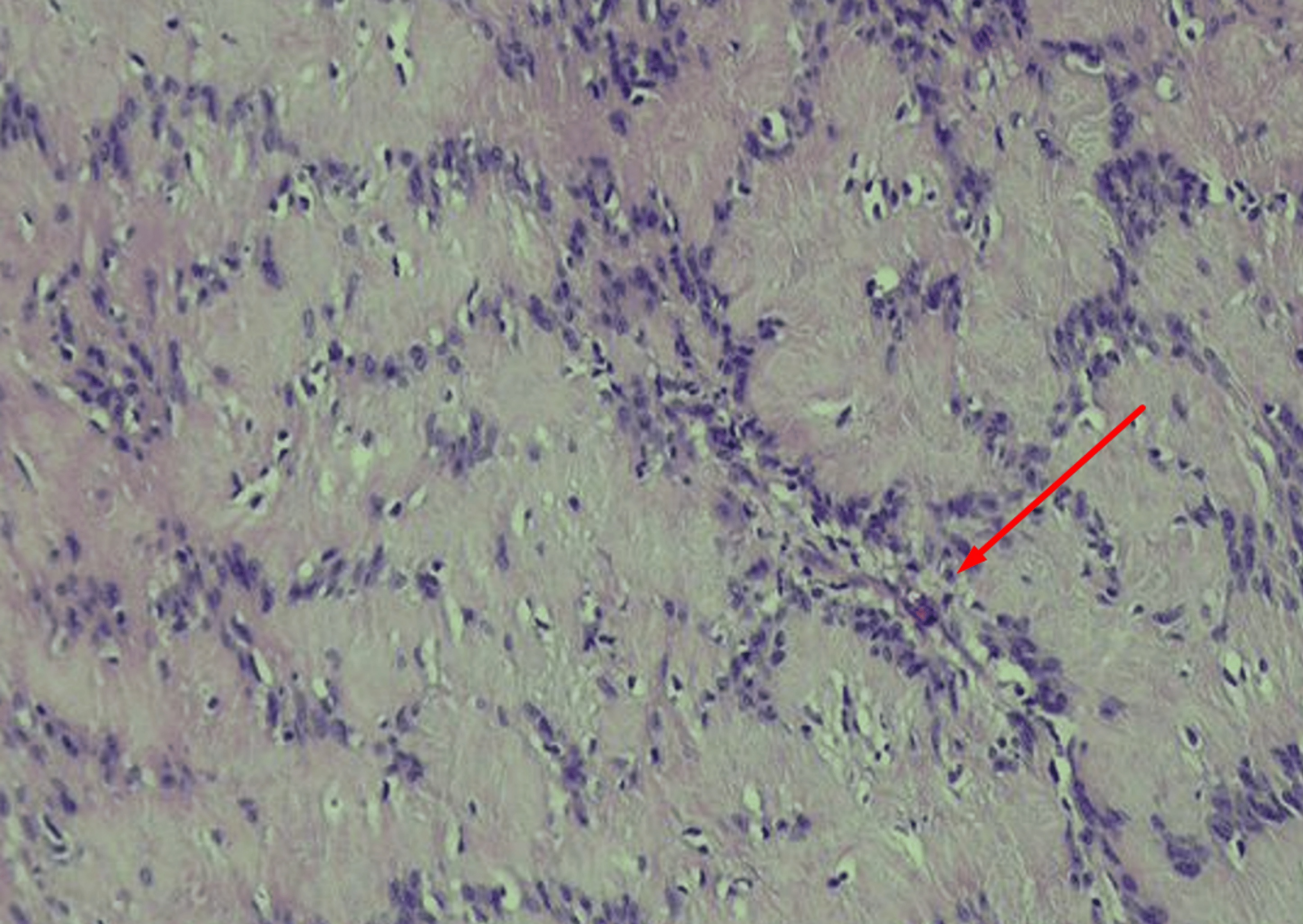
Histopathology depicting spindle cells with elongated-to-plump vesicular nuclei with occasional visible nucleoli (arrow).

The histopathology of the resected tumor showed biphasic regions, consisting of moderately cellular spindle-shaped tumor cells with alternating compact areas (Antoni A) and loosely arranged foci (Antoni B). The spindle cells are arranged in short fascicles with nuclear palisading (Verocay bodies) in Antoni A areas (Figure 6). These cells have plump and ovoid vesicular nuclei, indistinct nucleoli, and ill-defined eosinophilic cytoplasm. Immunohistochemical (IHC) staining was positive for SOX10 and negative for CD34. These features confirmed the diagnosis of intrathoracic schwannoma.

## Discussion

The etiology of a benign intrathoracic lesion causing HS is very rare and to date, only seven cases have definitively described intrathoracic schwannoma as the cause [4], with a few others in recent times [5-7].

The HS symptoms our patient experienced were trivial; as such, he did not seek any medical attention. Eliciting this feature allowed clinical correlation to the well-defined mass on the left superior posterior mediastinum on CXR. Preoperative identification is important as HS might also be a post-operative complication [5]. While post-operative recovery of HS has previously been described, the HS in our patient was persistent [3-5]. We hypothesize that a period of prolonged compression by the schwannoma likely resulted in this delayed recovery of the sympathetic chain, as seen in other modes of sympathetic chain injury [8].

HS typically carries with it the connotation of an aggressive malignant etiology. Given the active smoking status of our patient, lymphoma and lung malignancy (Pancoast tumor) were our initial considerations. However, the short duration of smoking (five years), a chronic HS, the absence of constitutional symptoms in a young man, and the well-circumscribed tumor on both CXR and CT thorax further argue against the diagnosis of lung malignancy [2]. It can be challenging to ascertain the diagnosis of an intrathoracic schwannoma based solely on a CT of the thorax, as variations in imaging are often observed. Small tumors are typically homogenous, while larger schwannomas are heterogenous due to cystic and hemorrhagic changes. Regardless, they are typically well circumscribed with occasional calcifications seen (10% of schwannomas) [3].

We offered surgical resection due to its large size and the presence of HS (likely a result of tumor compression on or tumor arising from the cervicothoracic sympathetic trunk). Of interest, schwannomas can co-exist with lung cancer, undergo malignant transformation, or even attain enormous sizes (>10 cm). If our patient’s injury had not occurred, this schwannoma would likely have been missed and progressed with time. This will eventually lead to adjacent organ compression, manifesting as tracheal deviation, dysphagia, dysphonia, or stridor. It might also lead to rib erosion and invasion into the spinal canal or pleural cavity. Fortunately, schwannoma typically occurs in a single site and recurrence is rare if complete resection is obtained. The exception, however, exists for patients with neurofibromatosis, as schwannoma may occur in multiple sites [3].

For non-operative candidates, the combination of an mTOR inhibitor and dasatinib may hold promise for the treatment of schwannomas [9]. Bevacizumab, a vascular endothelial growth factor (VEGF) receptor vaccine, might also be efficacious [10].

## Conclusions

Intrathoracic schwannoma may rarely be a cause of Horner syndrome. The diagnosis of intrathoracic schwannoma ought to be considered whenever a young individual presents with an incidental well-defined posterior mediastinal or paraspinal mass. As most tumors are often asymptomatic, early detection is typically based on routine chest radiography. Complete surgical resection is the cornerstone of treatment, whether open or video-assisted, and is dictated by tumor location, local extension, and, most importantly, tumor size. The prognosis is generally good.
